# Choroidal vascularity index as a biomarker of systemic inflammation in childhood Polyarteritis Nodosa and adenosine deaminase-2 deficiency

**DOI:** 10.1186/s12969-020-0417-3

**Published:** 2020-04-03

**Authors:** Ata BAYTAROĞLU, Sibel KADAYIFÇILAR, Abdullah AĞIN, Özge DELİKTAŞ, Selcan DEMİR, Yelda BİLGİNER, Jale KARAKAYA, Seza ÖZEN, Bora ELDEM

**Affiliations:** 1 Department of Ophthalmology, Aydın State Hospital, Aydın, Turkey; 2 Department of Ophthalmology, Hacettepe University School of Medicine, Ankara, Turkey; 3 Department of Ophthalmology, Patnos State Hospital, Ağrı, Turkey; 4 Department of Pediatrics, Pediatric Rheumatology Unit, Hacettepe University School of Medicine, Ankara, Turkey; 5 Department of Biostatistics, Hacettepe University School of Medicine, Ankara, Turkey

**Keywords:** PAN, DADA-2, Choroidal thickness, Choroidal vascularity index

## Abstract

**Background/purpose:**

To assess EDI-OCT (enhanced depth imaging optical coherence tomography) of choroid for inflammatory signs in children with polyarteritis nodosa (PAN) and adenosine deaminase-2 deficiency (DADA-2).

**Methods:**

In this cross-sectional study conducted between June 2017 and September 2018, we evaluated children diagnosed with PAN (*n* = 11) and DADA-2 (*n* = 4) and an age- and sex-matched control group (*n* = 15). Demographic and laboratory data were retrospectively analyzed from patient charts. Disease activity was assessed using the pediatric vasculitis activity score (PVAS). Choroidal images were obtained with spectral domain-OCT to measure choroidal thickness (ChT) at 5 points (750 and 1500 μm from the foveal center in the temporal and nasal quadrants and beneath the fovea), and to calculate the total subfoveal choroidal area (TCA), luminal area (LA), stromal area (SA), and the choroidal vascularity index (CVI).

**Results:**

The median (min-max) age was 8 (4–16) years in PAN patients, 6 (5–16) years in DADA-2 patients and 8 (8–10) years in control group at the OCT visit (*p* = 0.214). The ChT at 3 points and the TCA, LA, and SA were higher in children with both PAN and DADA-2 patients compared to those of the control group (*p* < 0.0001, *p* = 0.049, *p* = 0.007, p = 0.007, *p* = 0.006, *p* = 0.033, respectively). The CVI was similar in both groups. No association was observed between the OCT findings, PVAS, and the erythrocyte sedimentation rate, and serum leukocyte and C-reactive protein levels.

**Conclusion:**

Similar CVI scores were obtained from PAN and DADA2 patients under treatment and from healthy controls. Increased subfoveal ChT without any other signs of ocular involvement may suggest choroidal thickening as a sign of mild subclinical inflammation.

## Background

Polyarteritis nodosa (PAN) is a systemic necrotizing vasculitis affecting small or medium arteries. Patients present with negative antineutrophil cytoplasmic antibody (ANCA) serology and no evidence of glomerulonephritis [1, 2]. Unlike ANCA-associated vasculitides, pathogenesis of classic PAN remains unclear. PAN is rare, with incidence and prevalence rates of approximately 1/1000000 and 31/1000000, respectively in European adults. Notably, pediatric PAN is even rarer, and the largest pediatric PAN series (110 cases) published by Ozen [3] et al. reported that most PAN cases in children had multisystemic involvement. When Kussmaul and Maier first described PAN, nearly all cases of necrotizing vasculitis were classified as PAN however, Chapel Hill Classification criteria has now provided us with clear definitions for each vasculitis including PAN [1, 4]. Recently whole-exome sequencing has enabled the identification of monogenic diseases misdiagnosed as PAN. An example of this monogenic necrotizing vasculitis disease spectrum is deficiency of adenosine deaminase-2 (DADA2). In 2014, two studies, one by Navon Elkan [5] et al. and the other by Zhou Q [6] et al., reported an association between DADA2 and PAN, providing some insight to disease pathology. The authors highlighted the significance of the *cat eye syndrome chromosome region candidate 1 (CECR1)* gene, which encodes the ADA2 protein and its role in maintaining vascular integrity [6].

PAN and related monogenic diseases show a wide range of clinical manifestations including systemic symptoms (fever, myalgia, and weight loss) as well as cutaneous, gastrointestinal, renal, and neurological involvement [2, 7–10]. Ophthalmological manifestations, which occur in 5–40% of patients are relatively uncommon compared with involvement of other organ systems [2, 11]. The major ocular findings are similar to those occurring in other microvascular diseases and include retinal vasculitis, cotton-wool spots, and choroidopathy [7]. The involvement of retinal and choroidal arteries in patients with PAN was first reported by Goldsmith [11] in 1946. Hsu [12] et al. reported PAN-induced bilateral choroidal infarction. A recent report by Kostina-O’Neil [13] et al. describes a case of prominent choroidopathy and optic neuropathy presenting as early signs of PAN.

The choroid as the most vascular structure of the eye, consists of a capillary network and larger choroidal vessels. The choriocapillaris becomes wider and longer towards the equatorial region. In contrast to other capillary networks throughout the human body, the luminal diameter of these capillaries is significantly larger (approximately 20 μm at the macula and 18–50 μm at the periphery) [14]. Macula involves high density of photoreceptors, that results in a high metabolic demand at this region which is supplied primarily by choroid. Enhanced depth imaging is a commercially available non-invasive modification of standard optical coherence tomography with 6 μm axial resolution. It has enabled us to assess choroid which could not have been evaluated in detail before by methods such as fluorescein angiography or ultrasonography [15]. Importance of choroidal assessment has been evaluated in number of uveitides, such as birdshot chorioretinopathy, Vogt-Koyanagi-Harada (VKH) and Behçet’s diseases [16–18]. Choroidal thickness alterations have been noted in VKH and sarcoidosis even in subclinical conditions [19, 20]. Although choroidal thickness measurements must be obtained manually, previous studies have shown a dependable inter-observer reproducibility [21]. Like many other vascular structures, choroidal circulation is susceptible to many common pathologies, such as hyperlipidemia or hypertension, and to some physiological changes such as exercise or even ageing itself [22, 23]. Choroidal thickness has been shown to decrease approximately 1.2 μm each year of age [24]. Apart from these relatively small fluctuations, ChT has been shown to alter in inflammatory diseases even without a direct correlation with systemic disease activity [25]. Studies conducted on adult rheumatoid arthritis and SLE patients have both shown a 25% decrease in ChT due to chronic choroidal vascular inflammation [25, 26].

The aim of this article was to investigate possible subclinical ocular involvements in PAN and PAN-like vasculitides, which may precede sight threatening complications. Although choroidal findings have been reported in most cases with PAN-induced ocular manifestations, to the best of our knowledge, our study is the first to evaluate choroidal changes using enhanced depth imaging optical coherence tomography (EDI-OCT) and to investigate their possible association with clinical findings in children with PAN and DADA2 [11–13, 27].

## Methods

This study included 11 children with PAN, 4 with DADA2, and 15 age-matched healthy volunteers. The enrolled children were diagnosed with PAN according to the European League Against Rheumatism/Paediatric Rheumatology European Society/Paediatric Rheumatology International Trials Organization classification criteria for childhood PAN [28, 29]. All patients with DADA2 were diagnosed by defining the mutations of CECR1 by Sanger sequencing.

All children were evaluated by pediatric rheumatologists (SO and SD) and were referred to Ophthalmology Department for ocular examination. Demographic data, clinical manifestations, and biochemical parameters (the erythrocyte sedimentation rate, and serum levels of leukocyte and C-reactive protein) at the time of diagnosis were retrospectively analyzed from patient charts.

The pediatric vasculitis activity score (PVAS) [30] was calculated to evaluate the disease activity in children with PAN [31]. The following criteria were used for the diagnosis of active DADA2 disease: 1) elevated serum levels of acute phase reactants (APRs) or fever without evidence of infection and, 2) new symptoms related to DADA2. Ophthalmological assessment performed in all children included complete ocular examination with measurement of best corrected visual acuity (BCVA) with a Snellen chart. Anterior segment slit-lamp examination was performed to evaluate the conjunctiva, sclera, cornea, anterior chamber, iris and lens to identify signs of keratitis, episcleritis, scleritis, and/or uveitis and their sequelae at × 16 and × 40 magnification. The intraocular pressure (IOP) was measured using a Goldmann applanation tonometer and recorded in mmHg. Vitreus, retina, retinal vascular structures and optic disc were examined by dilated fundus examination with 90 diopter non-contact lens before EDI-OCT scan. Children with a history and/or finding of ocular disease unrelated to PAN or DADA2, and/or children with refractive errors > +/− 3.0 diopters were excluded from the study. OCT images of children with image quality index(Q) < 25 were also excluded. A spectral domain OCT device with EDI mode set to 870 nm (Heidelberg Spectralis, Heidelberg Engineering, Dossenheim, Germany) was used for measurements. The EDI-OCT images were obtained on horizontal OCT scans between 30° × 20° centered on the fovea. As choroidal thickness (ChT) is affected by the time of measurement, all measurements were performed between 09:00 and 11:00 a.m.

**Fig. 1:**
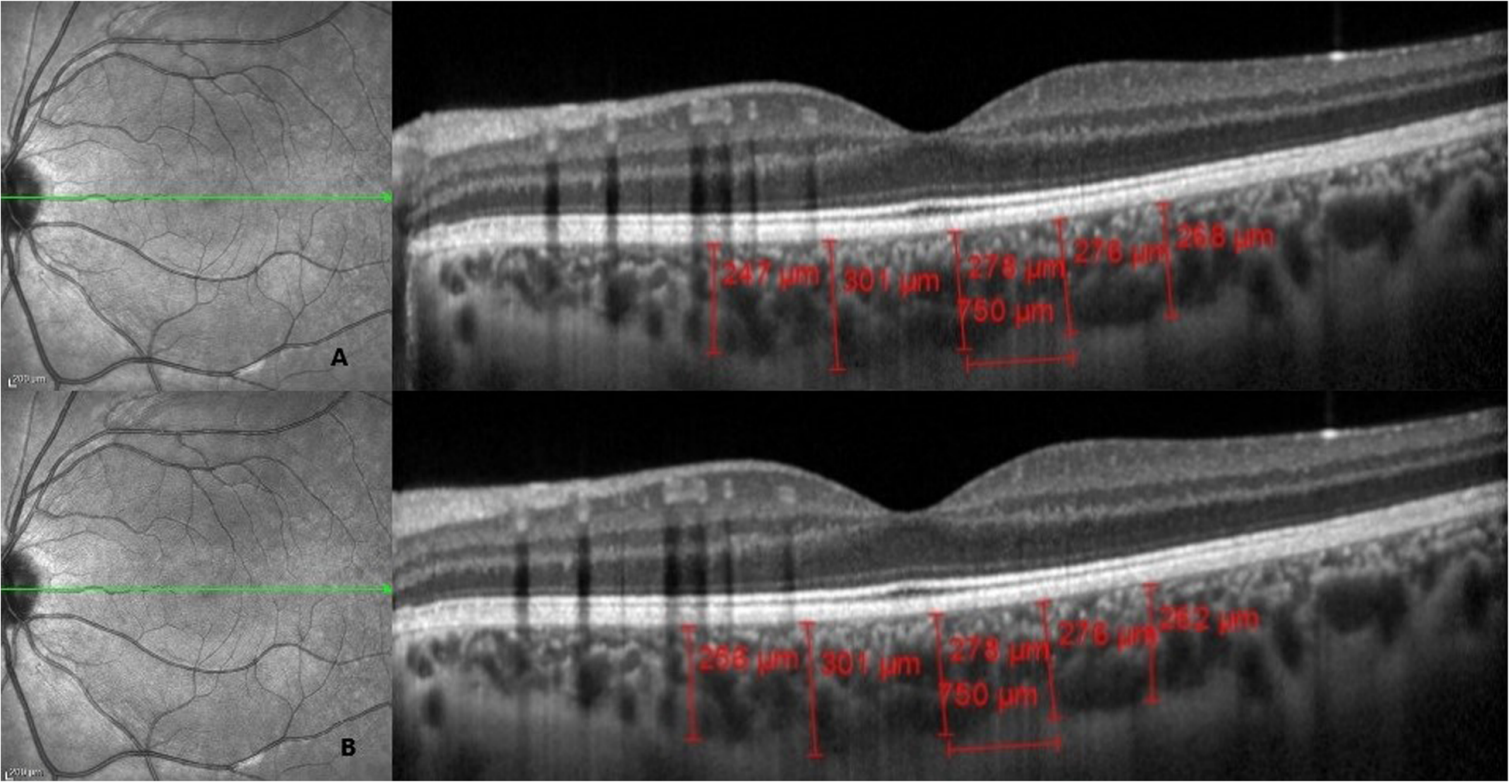
Assessment performed using enhanced depth imaging optical coherence tomography. Image showing choroidal thickness measurement shows the following: The cross-sectional image of the choroid is obtained using enhanced depth imaging scanning with optical coherence tomography. Measurements are obtained by 2 independent examiners at 5 locations using 750 μm intervals. **a** Measurements obtained by Examiner I. **b** Measurements obtained by Examiner II

Data from only one eye (left eye) of each patient and the control subjects were analyzed. Heidelberg Eye Explorer software (Heidelberg Engineering, Heidelberg, Germany) was used for measurements (Fig. 1). ChT was measured manually by 2 blinded observers from the outer surface of the retinal pigment epithelium to the lamina sclera at 5 points (750 and 1500 μm from the foveal center in the temporal (T) and nasal (N) quadrants and beneath the fovea). The mean of the 2 measurements was obtained for statistical analysis.

**Fig. 2:**
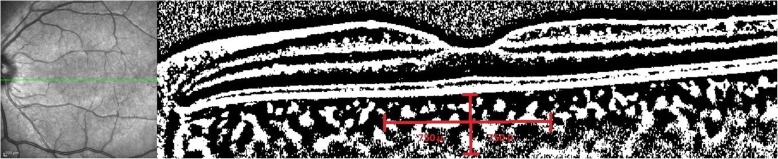
An optical coherence tomography section after binarization by ImageJ software*.* Black areas observed in the image represent the luminal area, and white areas represent the stromal area in choroidal tissue

ImageJ software (version 1.8.0_77, Bethesda, MD, USA http://imagej.nih.gov/ij/) was used to calculate the choroidal vascularity index (CVI). The images obtained with EDI-OCT were converted using the ImageJ software, and the scale was adjusted to 100 μm. A line measuring 750 μm was drawn from the foveal center, both nasally and temporally with a total length of 1500 μm. After this stage, the image type was changed to 8 bits and an automated local threshold was applied using the Niblack method [32] to convert all pixels from red, green, and blue to black and white. The number of pixels was measured using the histogram module, in which the total number of pixels could be measured using the count number. The number 0 represents luminal area (LA), and 255 represents stromal area (SA) when using the list option. We obtained the following values: Total choroidal area (TCA) = LA + SA and the CVI = LA÷TCA. A binarized OCT section is shown in Fig. 2.

### Statistical analysis

Statistical analysis was performed using the SPSS 21.0 software package (SPSS Inc. IBM, Armonk, NY, USA). Descriptive analyses were expressed using proportions, and medians (minimum and maximum values). Conformity of the variables to a normal distribution was investigated using visual (histogram and probability plots) and analytical methods (Kolmogorov-Smirnov tests). Kruskal Wallis test was performed to compare the differences among groups for non-parametric data, the Spearman test was used to calculate correlation coefficients and their significance. All data from either eye and for each point have been compared separately with Wilcoxon-Mann-Whitney test. A *p* value < 0.05 was considered statistically significant. Inter-examiner reproducibility of the ChT measurements was assessed by measuring the intraclass correlation coefficient (ICC).

## Results

This study group included 15 children (11 children with PAN, 4 children with DADA2). The median (minimum–maximum) ages at diagnosis were 4 (3–15) years, and ages at the OCT visit of the subjects were 8 (4–16) years. The median ages of healthy controls were 8 (8–10) years at the OCT visit. The median (minimum–maximum) duration of follow-up was 4 (2–6) years. The median (min-max) age was 8 (4–16) years in PAN patients, 6 (5–16) years in DADA-2 patients and 8 (8–10) years in control group at the OCT visit (*p* = 0.214). All children diagnosed with DADA2 were homozygous for a G47R mutation in the *CECR1* gene. At the time of diagnosis, the most common symptom was myalgia and arthralgia (92.8%), followed by fever and fatigue (85.7%), abdominal pain (85.7%), rash (78.5%), hypertension (21.4%), epididymoorchitis (14.2%), proteinuria (7.1%), posterior reversible encephalopathy syndrome (7.1%). At the last visit, only 1 child with DADA2 was confirmed as having active disease based on myalgia, arthralgia, vasculitic rash and elevated serum levels of APRs. The median (minimum–maximum) PVAS scores at the time of diagnosis and at the last visit were 5 (4–8) and 0 (0–4), respectively.

All children received corticosteroid treatment. All children with DADA2 received etanercept at the time of their last visit. Of the 11 children diagnosed with PAN, 7 received mycophenolate mofetil, 6 received also colchicine, and 2 received enalapril in addition to the primary treatment. Azathioprine and tocilizumab were administered to one child each.

**Fig. 3:**
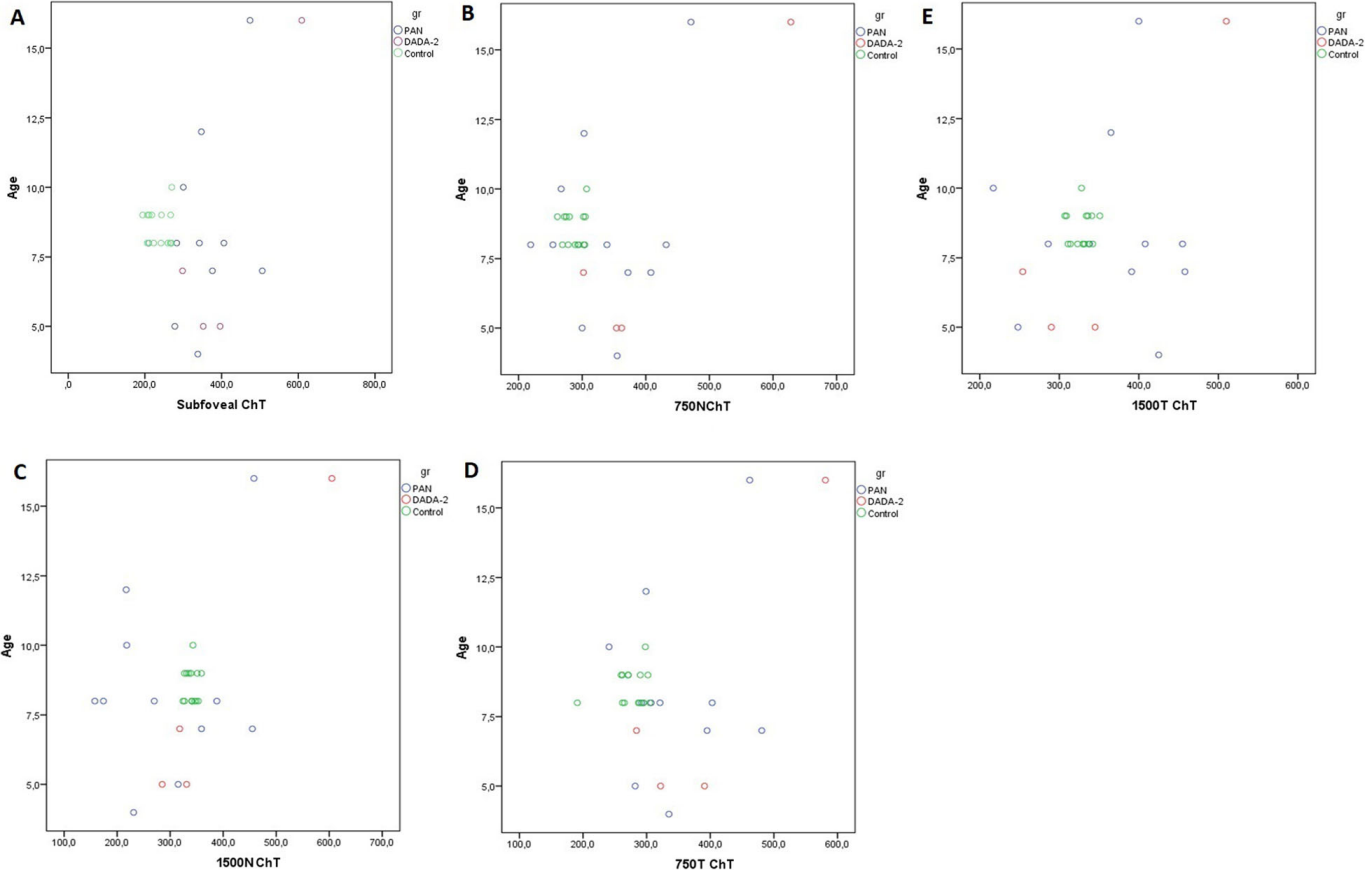
Plotted graph showing the relationship between choroidal thicknesses and age. **a**) Subfoveal ChT-Age **b**)750 N ChT-Age **c**)1500 N ChT-Age **d**)750 T ChT-Age **e**)1500 T ChT-Age

**Fig. 4:**
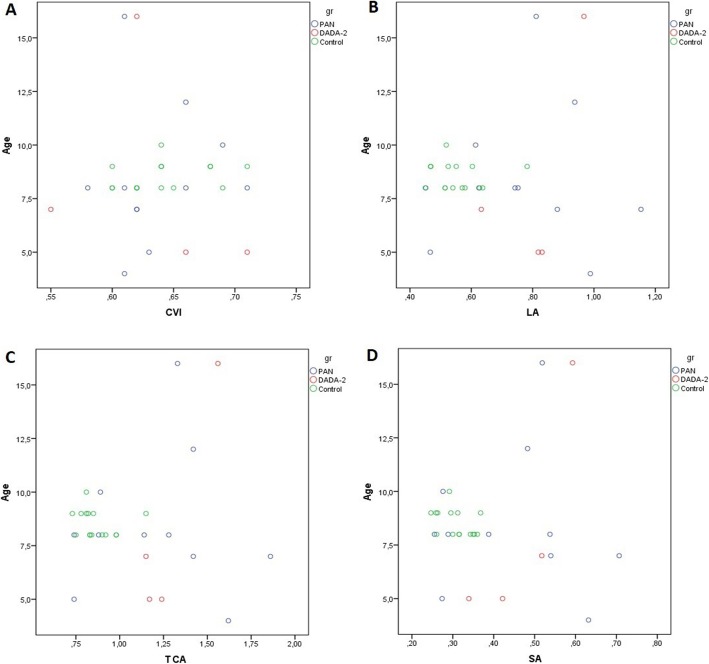
Plotted graph showing the relationship between the other OCT parameters and age. **a**) CVI-Age **b**) LA-Age **c**) TCA-Age **d**) SA-Age

Clinical characteristics of PAN and DADA2 patients at the time of diagnosis are highlighted separately in Table 1. The main clinical and laboratory findings observed in the enrolled children at the time of diagnosis and at the OCT visit are summarized comparatively in Table 2. The median BCVA was 20/25 (20/32–20/20). No patient had a history of any visual disturbances (e.g. red eye, floaters, blurred vision, photophobia, ocular pain) suggesting previous uveitis or any other ocular manifestation. Biomicroscopic anterior segment and dilated fundus examination did not reveal any pathology or sequela of a previous inflammation in any child. However, the choroid was significantly thicker in children with PAN and DADA-2 than in the control group at the subfoveal and at the 750 N and 750 T points (*p* < 0.0001, *p* = 0.049, *p* = 0.007, respectively). There was not any significant difference between the patients and healthy controls at 1500 N and 1500 T (*p* = 0.325, *p* = 0.278). The TCA, LA, and SA values in children with PAN and DADA-2 were greater than those in the control group (*p* = 0.007, *p* = 0.006, *p* = 0.033, respectively). No intergroup difference was observed in the CVI (*p* = 0.91). OCT assessments are summarized in Table 3. Post-hoc analysis results are highlighted in Table 4. The plotted graph in Fig. 3-4 shows the relationship between age and the other OCT parameters. Correlation analysis did not show a significant association between OCT findings, PVAS, and biochemical parameters. The inter-examiner ICC for ChT was > 0.90 (95% confidence interval 0.90–0.92) for all measurement points. There was no significant difference between the measurements of the study and fellow eyes. (*p* = 0.22).

**Table 1:** Clinical characteristics of PAN and DADA2 patients at the time of diagnosis

	PAN (*n* = 11)	DADA2 (*n* = 4)
Female/Male	6/5	1/3
Age at symptom onset, median (min-max)	4 (2–10)	3.5 (1.5–14)
Age at diagnosis median (min-max)	6 (3–12)	4 (3–15)
Age at the OCT visit median (min-max)	8 (4–16)	6 (5–16)
Constitutional symptoms, n (%)	8 (73%)	4 (100%)
Musculoskeletal symptoms, n (%)	10 (90%)	3 (75%)
Cutaneous involvement, n (%)	8 (73%)	3 (75%)
Hypertension, n (%)	0 (0%)	1 (25%)
Renal involvement, n (%)	0 (0%)	1 (25%)
Neurologic involvement, n (%)	1 (9%)	0 (0%)
Testis involvement, n (%)	1 (9%)	1 (25%)
Gastrointestinal system involvement, n (%)	9 (82%)	3 (75%)
Cardiovascular involvement, n (%)	0 (0%)	0 (0%)
Pulmonary involvement, n (%)	0 (0%)	0 (0%)
Medication n (%)		
Steroid	11 (100%)	4 (100%)
Biologic agent	1 (9%)	4 (100%)
DMARD	8 (83%)	0 (0%)

**Table 2:** The main characteristics of patients at the time of diagnosis and the last visit

	At the time of diagnosis	At the OCT visit
Constitutional symptoms, *n* (%)	12 (85.7)	0 (0)
Musculoskeletal symptoms, *n* (%)	13 (92.8)	1 (7.1)
Cutaneous involvement, *n* (%)	11 (78.5)	1 (7.1)
Hypertension, *n* (%)	3 (21.4)	0 (0)
Renal involvement, *n* (%)	1 (7.1)	0 (0)
Neurologic involvement, *n* (%)	1 (7.1)	0 (0)
Testis involvement, *n* (%)	2 (14.2)	0 (0)
Gastrointestinal system involvement, n (%)	12 (85.7)	0 (0)
Cardiovascular involvement, *n* (%)	0 (0)	0 (0)
Pulmonary involvement, *n* (%)	0 (0)	0 (0)
WBC^a^ count, ×10^3^/mm^3^, median (min-max)	15,200 (6500–29,200)	8800 (5200–12,600)
ESR^a^ (mm/h), median (min-max) (normal range 0–20)	45 (35–57)	17 (4–20)
CRP^a^ (mg/dl), median (min-max) (normal range 0–0.8)	17 (2.3–24.9)	1.3 (0.1–1.5)

^a^*CRP* C-reactive protein, *ESR* Erythrocyte sedimentation rate, *WBC* White blood cell

**Table 3:** Comparison of OCT findings between PAN and DADA2 patients and control group

Parameter	PAN patients (*n* = 11) Median (min-max)	DADA2 patients (*n* = 4) Median (min-max)	Control group (*n* = 15) Median (min-max)	*p*-value**
Subfoveal ChT	342 (201–506)	374 (298–609)	242 (194–270)	< 0.0001^*^
750 N ChT	339 (219–471)	358 (302–628)	293 (261–307)	0.049^*^
1500 N ChT	270 (158–458)	324.5 (285–605)	341 (324–359)	0.325
750 T ChT	321 (241–481)	356.5 (284–581)	287 (191–305)	0.007^*^
1500 ChT	391 (217–458)	317.5 (254–510)	330 (307–351)	0.278
Total Subfoveal Choroidal Area	1.28 (0.74–1.86)	1.2 (1.15–1.56)	0.83 (0.73–1.15)	0.007^*^
Luminal Area	0.75 (0.45–1.15)	0.82 (0.63–0.96)	0.54 (0.45–0.78)	0.006^*^
Stromal Area	0.48 (0.25–0.70)	0.46 (0.33–0.59)	0.31 (0.24–0.36)	0.033^*^
CVI	0.62 (0.58–0.71)	0.64 (0.55–0.71)	0.64 (0.60–0.71)	0.910

^*^, statistically significant

**Table 4:** Post-hoc analysis results for statistically significant variables in PAN, DADA2 and control group

Parameter	PAN- Control Group (*p* value)	DADA2–Control Group (*p* value)	PAN- DADA2 Group (*p* value)
Subfoveal ChT	< 0.0001^*^	< 0.001^*^	0.504
750 N ChT	0.11	0.024^*^	0.277
750 T ChT	0.006^*^	0.02^*^	0.697
Total Subfoveal Choroidal Area	0.013^*^	0.01^*^	0.425
Luminal Area	0.012^*^	0.008^*^	0.384
Stromal Area	0.043^*^	0.029^*^	0.471

^*^, statistically significant

## Discussion

Our findings in this small series of PAN and DADA2 showed a thicker choroidal tissue at three points (Subfoveal, 750 N, 750 T) compared to healthy subjects, which may reflect the systemic inflammation in these children. Lack of such difference at 1500 N and 1500 T might be attributable to natural thinning of choroid nasally and thickening temporally [33]. Few studies in current literature mentions choroidal vascular involvement in PAN-like disease spectrum, and these studies reveal choroidal involvement in severe cases [12, 13]. Central retinal occlusion, panuveitis and oculomotor nerve palsy, have been previously reported in the spectrum of PAN [27]. Akova [34] et al. previously reported ocular inflammation as one of the earliest signs in adult PAN. Our study is the first report of a possible choroidal involvement even in mild inflammatory status in this disease spectrum. Further analysis also revealed that this expansion is homogenous in terms of affected choroidal structures. Both luminal and stromal areas have been found to be thicker in PAN and DADA2 group (0.76 ± 0.19 mm^2^ and 0.44 ± 0.14 mm^2^), CVI did not show any significant difference between two groups.

Choroidal expansion may occur (similar to choroidal infarction) secondary to systemic or local inflammation of the choroidal vascular network. Though we found choroid to be thicker, we could not demonstrate correlations with systemic inflammatory markers and choroidal findings in our study. Studies on rheumatic diseases causing both systemic and local ocular inflammation, such as Behçet’s disease, have reported increased ChT secondary to increased pro-inflammatory cytokine levels, primarily in the acute phase of the disease. However, effacement replaces expansion as the disease progresses and chronic vascular injury settles [35–38]. Onal [39] et al. reported choroidal stromal expansion without any association with ChT in patients with active Behçet’s uveitis. It is noteworthy that in our series, none of the patients had an active uveitis or any other ocular involvement and only one patient had clinically active DADA2, suggesting that even under a successful treatment regimen, a mild subclinical inflammation may still exist. Although two patients were on enalapril due to a previous renal involvement, we did not regard this as a confounding factor because of their euvolemic and normotensive state. As for other mainstay treatment agents that might have altered choroidal thicknesses, both glucocorticoids and etanercept have been shown to decrease endothelial damage, sustain blood-retinal barrier and prevent further leakage thus expected to result in a decreasing effect on ChT [40–42].

The CVI is a novel parameter to evaluate changes in the choroidal structure and shows the ratio of LA to TCA [43, 44]. Increased LA is observed in polypoidal choroidal vasculopathy and central serous chorioretinopathy secondary to expansion of the intravascular space and leakage from dilated choroidal vessels [45, 46]. External components that represent SA are mainly composed of connective tissue elements and are affected by ongoing autoinflammatory processes causing stromal edema [47, 48]. Agarwal [44] et al. reported that increased LA and SA were more commonly observed during an acute attack than during convalescence secondary to ocular inflammation in patients with tubercular multifocal serpiginoid choroiditis. Kim [49] et al. reported an increase in the CVI and ChT during an attack of HLA-B27 associated uveitis; however, LA and SA were not evaluated separately in this study. LA measurements are important because although the LA is 60–65% of the TCA, it is not affected as much as TCA by factors such as axial length, refractive errors, IOP, and diurnal variations [50, 51]. It has also been suggested that in patients with a higher TCA, prolonged compression by LA causes stromal atrophy. In contrast, a higher SA with a low CVI is observed in patients with chronic inflammatory diseases and diseases that cause choroidal hyperpermeability.

In the current study, both, the LA and SA were increased in the study group compared with the control group. Primary endothelial dysfunction occurs secondary to primary inflammatory vasculitis and the action of pro-inflammatory cytokines or antibodies across this disease spectrum [52, 53]. The increase in both the LA and SA but a stable CVI observed in current study group can be attributed to the fact that PAN and DADA2 individually cause microaneurysms and increased vascular permeability owing to vasculitis in LA, as well as systemic inflammation causing an expanded SA. Increased ChT is attributed to changes in both LA and SA.

Limitations of this study include a relatively small sample size of children with PAN and DADA-2. The study is cross-sectional, we do not have OCT data before the start of treatment. Although accompanying inflammation causes choroidal stromal expansion, PAN-like conditions primarily affect the luminal aspects of vascular structures. Thus, further evaluation based on quantitative analysis of inflammatory markers will provide a better understanding of the association between choroidal structural changes and systemic conditions. Further research with more subjects, choroidal assessment at the time of diagnosis and a longer follow-up period is required to determine the value of the role of ophthalmic assessments in monitoring disease activity.

## Conclusions

This study is the first case series describing choroidal involvement in children with the PAN and DADA2. The choroid was observed to be thicker in the PAN and DADA-2 group with an increase in the LA, SA and TCA.

## Data Availability

The datasets used and/or analyzed during the current study are available from the corresponding author on reasonable request.

## Abbreviations

APRs: Acute phase reactants
ChT: Choroidal thickness
CVI: Choroidal vascularity index
DADA-2: Adenosine deaminase-2 deficiency
LA: Luminal area
OCT: Optical coherence tomography
PAN: Polyarteritis nodosa
PVAS: Pediatric vasculitis activity score
SA: Stromal area
TCA: Total subfoveal choroidal area

## References

[1] Jennette JC, Falk RJ, Bacon PA, Basu N, Cid MC, Ferrario F, Flores-Suarez LF, Gross WL, Guillevin L, Hagen EC, Hoffman GS, Jayne DR, Kallenberg CG, Lamprecht P, Langford CA, Luqmani RA, Mahr AD, Matteson EL, Merkel PA, Ozen S, Pusey CD, Rasmussen N, Rees AJ, Scott DG, Specks U, Stone JH, Takahashi K, Watts RA (2013). 2012 revised international Chapel Hill consensus conference nomenclature of Vasculitides. Arthritis Rheum.

[2] Hernandez-Rodriguez J, Alba MA, Prieto-Gonzalez S, Cid MC (2014). Diagnosis and classification of polyarteritis nodosa. J Autoimmun.

[3] Ozen S, Anton J, Arisoy N, Bakkaloglu A, Besbas N, Brogan P, Garcia-Consuegra J, Dolezalova P, Dressler F, Duzova A, Ferriani VP, Hilario MO, Ibanez-Rubio M, Kasapcopur O, Kuis W, Lehman TJ, Nemcova D, Nielsen S, Oliveira SK, Schikler K, Sztajnbok F, Terreri MT, Zulian F, Woo P (2004). Juvenile polyarteritis: results of a multicenter survey of 110 children. J Pediatr.

[4] Matteson EL, Kluge FJ (2003). Think clearly, be sincere, act calmly: Adolf Kussmaul (February 22, 1822-may 28, 1902) and his relevance to medicine in the 21st century. Curr Opin Rheumatol.

[5] Navon Elkan P, Pierce SB, Segel R, Walsh T, Barash J, Padeh S, Zlotogorski A, Berkun Y, Press JJ, Mukamel M, Voth I, Hashkes PJ, Harel L, Hoffer V, Ling E, Yalcinkaya F, Kasapcopur O, Lee MK, Klevit RE, Renbaum P, Weinberg-Shukron A, Sener EF, Schormair B, Zeligson S, Marek-Yagel D, Strom TM, Shohat M, Singer A, Rubinow A, Pras E, Winkelmann J, Tekin M, Anikster Y, King MC, Levy-Lahad E (2014). Mutant adenosine deaminase 2 in a polyarteritis nodosa vasculopathy. N Engl J Med.

[6] Zhou Q, Yang D, Ombrello AK, Zavialov AV, Toro C, Zavialov AV, Stone DL, Chae JJ, Rosenzweig SD, Bishop K, Barron KS, Kuehn HS, Hoffmann P, Negro A, Tsai WL, Cowen EW, Pei W, Milner JD, Silvin C, Heller T, Chin DT, Patronas NJ, Barber JS, Lee CC, Wood GM, Ling A, Kelly SJ, Kleiner DE, Mullikin JC, Ganson NJ, Kong HH, Hambleton S, Candotti F, Quezado MM, Calvo KR, Alao H, Barham BK, Jones A, Meschia JF, Worrall BB, Kasner SE, Rich SS, Goldbach-Mansky R, Abinun M, Chalom E, Gotte AC, Punaro M, Pascual V, Verbsky JW, Torgerson TR, Singer NG, Gershon TR, Ozen S, Karadag O, Fleisher TA, Remmers EF, Burgess SM, Moir SL, Gadina M, Sood R, Hershfield MS, Boehm M, Kastner DL, Aksentijevich I (2014). Early-onset stroke and vasculopathy associated with mutations in ADA2. N Engl J Med.

[7] Pagnoux C, Seror R, Henegar C, Mahr A, Cohen P, Le Guern V, Bienvenu B, Mouthon L, Guillevin L, French Vasculitis Study G (2010). Clinical features and outcomes in 348 patients with polyarteritis nodosa: a systematic retrospective study of patients diagnosed between 1963 and 2005 and entered into the French Vasculitis study group database. Arthritis Rheum.

[8] Caorsi R, Penco F, Grossi A, Insalaco A, Omenetti A, Alessio M, Conti G, Marchetti F, Picco P, Tommasini A, Martino S, Malattia C, Gallizi R, Podda RA, Salis A, Falcini F, Schena F, Garbarino F, Morreale A, Pardeo M, Ventrici C, Passarelli C, Zhou Q, Severino M, Gandolfo C, Damonte G, Martini A, Ravelli A, Aksentijevich I, Ceccherini I, Gattorno M (2017). ADA2 deficiency (DADA2) as an unrecognised cause of early onset polyarteritis nodosa and stroke: a multicentre national study. Ann Rheum Dis.

[9] Nanthapisal S, Murphy C, Omoyinmi E, Hong Y, Standing A, Berg S, Ekelund M, Jolles S, Harper L, Youngstein T, Gilmour K, Klein NJ, Eleftheriou D, Brogan PA (2016). Deficiency of adenosine Deaminase type 2: a description of phenotype and genotype in fifteen cases. Arthritis Rheumatol.

[10] Ozen S, Bilginer Y, Batu ED, Taskiran E, Ozkara HA, Unal S, Guleray N, Erden A, Karadag O, Gumruk F, Cetin M, Sonmez HE, Ayvaz DC, Tezcan I. A monogenic disease with a variety of phenotypes: deficiency of adenosine deaminase 2 subtitle: deficiency of adenosine deaminase 2. J Rheumatol. 2019;47(1):117–25. 10.3899/jrheum.181384. 10.3899/jrheum.181384 31043544

[11] Goldsmith J (1946). Periarteritis nodosa with involvement of the choroidal and retinal arteries. Am J Ophthalmol.

[12] Hsu CT, Kerrison JB, Miller NR, Goldberg MF (2001). Choroidal infarction, anterior ischemic optic neuropathy, and central retinal artery occlusion from polyarteritis nodosa. Retina.

[13] Kostina-O'Neil Y, Jirawuthiworavong GV, Podell DN, Lesser RL (2007). Choroidal and optic nerve infarction in hepatitis C-associated polyarteritis nodosa. J Neuroophthalmol.

[14] Coscas G, Lupidi M, Coscas F, Chhablani J, Cagini C (2018). Optical coherence tomography angiography in healthy subjects and diabetic patients. Ophthalmologica.

[15] Kim JS, Knickelbein JE, Jaworski L, Kaushal P, Vitale S, Nussenblatt RB, Sen HN (2016). Enhanced depth imaging optical coherence tomography in uveitis: an Intravisit and Interobserver reproducibility study. Am J Ophthalmol.

[16] da Silva FT, Sakata VM, Nakashima A, Hirata CE, Olivalves E, Takahashi WY, Costa RA, Yamamoto JH (2013). Enhanced depth imaging optical coherence tomography in long-standing Vogt-Koyanagi-Harada disease. Br J Ophthalmol.

[17] Kim M, Kim H, Kwon HJ, Kim SS, Koh HJ, Lee SC (2013). Choroidal thickness in Behcet's uveitis: an enhanced depth imaging-optical coherence tomography and its association with angiographic changes. Invest Ophthalmol Vis Sci.

[18] Young M, Fallah N, Forooghian F (2015). Choroidal degeneration in birdshot chorioretinopathy. Retina.

[19] Ishibazawa A, Kinouchi R, Minami Y, Katada A, Yoshida A (2014). Recurrent Vogt-Koyanagi-Harada disease with sensorineural hearing loss and choroidal thickening. Int Ophthalmol.

[20] Gungor SG, Akkoyun I, Reyhan NH, Yesilirmak N, Yilmaz G (2014). Choroidal thickness in ocular sarcoidosis during quiescent phase using enhanced depth imaging optical coherence tomography. Ocul Immunol Inflamm.

[21] Rahman W, Chen FK, Yeoh J, Patel P, Tufail A, Da Cruz L (2011). Repeatability of manual subfoveal choroidal thickness measurements in healthy subjects using the technique of enhanced depth imaging optical coherence tomography. Invest Ophthalmol Vis Sci.

[22] Manjunath V, Taha M, Fujimoto JG, Duker JS (2010). Choroidal thickness in normal eyes measured using cirrus HD optical coherence tomography. Am J Ophthalmol.

[23] Tittl M, Maar N, Polska E, Weigert G, Stur M, Schmetterer L (2005). Choroidal hemodynamic changes during isometric exercise in patients with inactive central serous chorioretinopathy. Invest Ophthalmol Vis Sci.

[24] Margolis R, Spaide RF (2009). A pilot study of enhanced depth imaging optical coherence tomography of the choroid in normal eyes. Am J Ophthalmol.

[25] Duru N, Altinkaynak H, Erten S, Can ME, Duru Z, Ugurlu FG, Cagil N (2016). Thinning of Choroidal thickness in patients with rheumatoid arthritis unrelated to disease activity. Ocul Immunol Inflamm.

[26] Altinkaynak H, Duru N, Uysal BS, Erten S, Kurkcuoglu PZ, Yuksel N, Duru Z, Cagil N (2016). Choroidal thickness in patients with systemic lupus Erythematosus analyzed by spectral-domain optical coherence tomography. Ocul Immunol Inflamm.

[27] Sahin S, Adrovic A, Barut K, Ugurlu S, Turanli ET, Ozdogan H, Kasapcopur O (2018). Clinical, imaging and genotypical features of three deceased and five surviving cases with ADA2 deficiency. Rheumatol Int.

[28] Ozen S, Pistorio A, Iusan SM, Bakkaloglu A, Herlin T, Brik R, Buoncompagni A, Lazar C, Bilge I, Uziel Y, Rigante D, Cantarini L, Hilario MO, Silva CA, Alegria M, Norambuena X, Belot A, Berkun Y, Estrella AI, Olivieri AN, Alpigiani MG, Rumba I, Sztajnbok F, Tambic-Bukovac L, Breda L, Al-Mayouf S, Mihaylova D, Chasnyk V, Sengler C, Klein-Gitelman M, Djeddi D, Nuno L, Pruunsild C, Brunner J, Kondi A, Pagava K, Pederzoli S, Martini A, Ruperto N, Paediatric Rheumatology International Trials O (2010) EULAR/PRINTO/PRES criteria for Henoch-Schonlein purpura, childhood polyarteritis nodosa, childhood Wegener granulomatosis and childhood Takayasu arteritis: Ankara 2008. Part II: final classification criteria. Ann Rheum Dis 69 (5):798–806. doi:10.1136/ard.2009.116657. 10.1136/ard.2009.116657 20413568

[29] Ozen S, Ruperto N, Dillon MJ, Bagga A, Barron K, Davin JC, Kawasaki T, Lindsley C, Petty RE, Prieur AM, Ravelli A, Woo P (2006). EULAR/PReS endorsed consensus criteria for the classification of childhood vasculitides. Ann Rheum Dis.

[30] Dolezalova P, Price-Kuehne FE, Ozen S, Benseler SM, Cabral DA, Anton J, Brunner J, Cimaz R, O'Neil KM, Wallace CA, Wilkinson N, Eleftheriou D, Demirkaya E, Bohm M, Krol P, Luqmani RA, Brogan PA (2013). Disease activity assessment in childhood vasculitis: development and preliminary validation of the Paediatric Vasculitis activity score (PVAS). Ann Rheum Dis.

[31] Mukhtyar C, Lee R, Brown D, Carruthers D, Dasgupta B, Dubey S, Flossmann O, Hall C, Hollywood J, Jayne D, Jones R, Lanyon P, Muir A, Scott D, Young L, Luqmani RA (2009). Modification and validation of the Birmingham Vasculitis activity score (version 3). Ann Rheum Dis.

[32] Liu S, Du L, Zhou Q, Zhang Q, Hu K, Qi J, Liang L, Zhou C, Kijlstra A, Yang P. The Choroidal vascularity index decreases and Choroidal thickness increases in Vogt-Koyanagi-Harada disease patients during a recurrent anterior uveitis attack. Ocul Immunol Inflamm. 2017;26(8):1237–43. 10.1080/09273948.2017.1343357. 10.1080/09273948.2017.1343357 28914578

[33] Jin P, Zou H, Zhu J, Xu X, Jin J, Chang TC, Lu L, Yuan H, Sun S, Yan B, He J, Wang M, He X (2016). Choroidal and retinal thickness in children with different refractive status measured by swept-source optical coherence tomography. Am J Ophthalmol.

[34] Akova YA, Jabbur NS, Foster CS (1993). Ocular presentation of polyarteritis nodosa. Clinical course and management with steroid and cytotoxic therapy. Ophthalmology.

[35] Chung YR, Cho EH, Jang S, Lee SY, Lee ES, Lee K (2018). Choroidal thickness indicates subclinical ocular and systemic inflammation in eyes with Behcet disease without active inflammation. Korean J Ophthalmol.

[36] Ishikawa S, Taguchi M, Muraoka T, Sakurai Y, Kanda T, Takeuchi M (2014). Changes in subfoveal choroidal thickness associated with uveitis activity in patients with Behcet's disease. Br J Ophthalmol.

[37] Kim SW, Oh J, Kwon SS, Yoo J, Huh K (2011). Comparison of choroidal thickness among patients with healthy eyes, early age-related maculopathy, neovascular age-related macular degeneration, central serous chorioretinopathy, and polypoidal choroidal vasculopathy. Retina.

[38] Bicer T, Celikay O, Kosker M, Alp MY, Ozisler C, Yesilyurt A, Kucuk Bicer B, Gurdal C (2017). Retinal and Choroidal thickness in adult patients with familial Mediterranean fever. Ophthalmic Epidemiol.

[39] Onal S, Uludag G, Oray M, Mengi E, Herbort CP, Akman M, Metin MM, Koc Akbay A, Tugal-Tutkun I (2018). Quantitative analysis of structural alterations in the choroid of patients with active Behcet uveitis. Retina.

[40] Fauser S, Kalbacher H, Alteheld N, Koizumi K, Krohne TU, Joussen AM (2004). Pharmacokinetics and safety of intravitreally delivered etanercept. Graefes Arch Clin Exp Ophthalmol.

[41] Yang S, Zhang L (2004). Glucocorticoids and vascular reactivity. Curr Vasc Pharmacol.

[42] Braga J, Rothwell R, Oliveira M, Rodrigues D, Fonseca S, Varandas R, Ribeiro L (2019). Choroid thickness profile in patients with lupus nephritis. Lupus.

[43] Egawa M, Mitamura Y, Sano H, Akaiwa K, Niki M, Semba K, Sonoda S, Sakamoto T (2015). Changes of choroidal structure after treatment for primary intraocular lymphoma: retrospective, observational case series. BMC Ophthalmol.

[44] Agarwal A, Agrawal R, Khandelwal N, Invernizzi A, Aggarwal K, Sharma A, Singh R, Bansal R, Sharma K, Singh N, Gupta V (2018). Choroidal structural changes in tubercular multifocal Serpiginoid Choroiditis. Ocul Immunol Inflamm.

[45] Agrawal R, Chhablani J, Tan KA, Shah S, Sarvaiya C, Banker A (2016). Choroidal vascularity index in central serous Chorioretinopathy. Retina.

[46] Bakthavatsalam M, Ng DS, Lai FH, Tang FY, Brelen ME, Tsang CW, Lai TY, Cheung CY (2017). Choroidal structures in polypoidal choroidal vasculopathy, neovascular age-related maculopathy, and healthy eyes determined by binarization of swept source optical coherence tomographic images. Graefes Arch Clin Exp Ophthalmol.

[47] Chan CC (2003). Molecular pathology of primary intraocular lymphoma. Trans Am Ophthalmol Soc.

[48] Read RW, Zamir E, Rao NA (2002). Neoplastic masquerade syndromes. Surv Ophthalmol.

[49] Kim M, Kim RY, Park YH. Choroidal Vascularity Index and Choroidal Thickness in Human Leukocyte Antigen-B27-Associated Uveitis. Ocul Immunol Inflamm. 2018;27(8):1280–7. 10.1080/09273948.2018.1530364. 10.1080/09273948.2018.1530364 30285514

[50] Agrawal R, Gupta P, Tan KA, Cheung CM, Wong TY, Cheng CY (2016). Choroidal vascularity index as a measure of vascular status of the choroid: measurements in healthy eyes from a population-based study. Sci Rep.

[51] Sonoda S, Sakamoto T, Yamashita T, Uchino E, Kawano H, Yoshihara N, Terasaki H, Shirasawa M, Tomita M, Ishibashi T (2015). Luminal and stromal areas of choroid determined by binarization method of optical coherence tomographic images. Am J Ophthalmol.

[52] Filer AD, Gardner-Medwin JM, Thambyrajah J, Raza K, Carruthers DM, Stevens RJ, Liu L, Lowe SE, Townend JN, Bacon PA (2003). Diffuse endothelial dysfunction is common to ANCA associated systemic vasculitis and polyarteritis nodosa. Ann Rheum Dis.

[53] Chanseaud Y, Garcia de la Pena-Lefebvre P, Guilpain P, Mahr A, Tamby MC, Uzan M, Guillevin L, Boissier MC, Mouthon L (2003). IgM and IgG autoantibodies from microscopic polyangiitis patients but not those with other small- and medium-sized vessel vasculitides recognize multiple endothelial cell antigens. Clin Immunol.

